# Prognostic and predictive importance of the estrogen receptor coactivator AIB1 in a randomized trial comparing adjuvant letrozole and tamoxifen therapy in postmenopausal breast cancer: the Danish cohort of BIG 1-98

**DOI:** 10.1007/s10549-017-4416-0

**Published:** 2017-08-01

**Authors:** S. Alkner, M.-B. Jensen, B. B. Rasmussen, P.-O. Bendahl, M. Fernö, L. Rydén, H. Mouridsen, Danish Breast Cancer Cooperative Group

**Affiliations:** 1 Department of Clinical Sciences Lund, Oncology and Pathology, Skane University Hospital, Lund University, Lund, Sweden; 2 Danish Breast Cancer Cooperative Group, Department of Oncology, Rigshospitalet - Copenhagen University Hospital, Copenhagen, Denmark; 3 Department of Pathology, Herlev University Hospital, Herlev, Denmark; 4 Department of Clinical Sciences Lund, Oncology and Pathology, Faculty of Medicine, Lund University, Lund, Sweden; 5 Department of Clinical Sciences Lund, Surgery, Skane University Hospital, Lund University, Lund, Sweden; 6 Skane Clinic of Oncology, Skane University Hospital Lund, SE 222 41 Lund, Sweden

**Keywords:** Breast cancer, Tamoxifen, Aromatase inhibitor, Amplified in breast cancer 1 (AIB1), BIG 1-98, Randomized trial

## Abstract

**Purpose:**

To evaluate the estrogen receptor coactivator amplified in breast cancer 1 (AIB1) as a prognostic marker, as well as a predictive marker for response to adjuvant tamoxifen and/or aromatase inhibitors, in early estrogen receptor-positive breast cancer.

**Method:**

AIB1 was analyzed with immunohistochemistry in tissue microarrays of the Danish subcohort (*N* = 1396) of the International Breast Cancer Study Group’s trial BIG 1-98 (randomization between adjuvant tamoxifen versus letrozole versus the sequence of the two drugs).

**Results:**

Forty-six percent of the tumors had a high AIB1 expression. In line with previous studies, AIB1 correlated to a more aggressive tumor-phenotype (*HER2* amplification and a high malignancy grade). High AIB1 also correlated to higher estrogen receptor expression (80–100 vs. 1–79%), and ductal histological type. High AIB1 expression was associated with a poor disease-free survival (univariable: hazard ratio 1.35, 95% confidence interval 1.12–1.63. Multivariable: hazard ratio 1.29, 95% confidence interval 1.06–1.58) and overall survival (univariable: hazard ratio 1.34, 95% confidence interval 1.07–1.68. Multivariable: hazard ratio 1.25, 95% confidence interval 0.99–1.60). *HER2* did not seem to modify the prognostic effect of AIB1. No difference in treatment effect between tamoxifen and letrozole in relation to AIB1 was found.

**Conclusions:**

In a subset of the large international randomized trial BIG 1-98, we confirm AIB1 to be a strong prognostic factor in early breast cancer. Hence, although tumor AIB1 expression does not seem to be useful for the choice of tamoxifen versus an aromatase inhibitor in postmenopausal endocrine-responsive breast cancer, AIB1 is an interesting target for new anti-cancer therapies and further investigations of this biomarker is warranted.

## Introduction

One of the biggest challenges in today’s breast cancer care is to adjust adjuvant treatment, according to both tumor and patient characteristics, for optimal treatment aimed at improved prognosis. Although both tamoxifen and aromatase inhibitors have been shown to improve survival in estrogen receptor-positive breast cancer, the disease will recur in many patients despite adjuvant treatment (7–9% breast cancer recurrences five years after randomization in BIG-98 [1]). Moreover, in the metastatic setting most tumors eventually develop resistance to the given treatment. Hence, further studies of potential prognostic and predictive biomarkers are essential in order to optimize and individualize breast cancer treatment.

An interesting biomarker in relation to endocrine treatment is AIB1 (amplified in breast cancer 1). AIB1 belongs to the p160 steroid receptor coactivator family and interacts with the estrogen receptor in a ligand-dependent manner to enhance transcription [2, 3]. However, it has also been shown to interact with other transcription factors and signaling pathways inducing hormone-independent proliferation [4–6]. In human breast cancer, AIB1 correlates with factors indicating a more aggressive phenotype (*HER2* amplification, DNA non-diploidy, a high tumor grade, a high S-phase fraction, and high Ki67) [5, 7–10]. Several studies have also indicated AIB1 to be associated with endocrine treatment effect [5, 7, 8, 11–13], although results have not been unanimous. We have previously shown AIB1 to be both a prognostic marker and a predictive marker for adjuvant tamoxifen in a randomized trial of premenopausal women receiving adjuvant tamoxifen for 2 years versus control, and in independent cohorts [9, 10, 14]. These data were extended also to postmenopausal patients in an independent randomized trial of adjuvant tamoxifen versus control [15]. Women with estrogen receptor-positive breast cancer expressing high levels of AIB1 have a worse prognosis, but respond well to tamoxifen. The prognosis of women with low tumor expression of AIB1, on the other hand, is not further improved by tamoxifen, although early on they have a better prognosis. However, previous studies of AIB1 in relation to aromatase inhibitors are very sparse, and its predictive value for treatment with aromatase inhibitors has not been evaluated in any larger clinical trial. If patients with low tumor expression of AIB1 would still benefit from aromatase inhibitors, AIB1 might be a predictive marker for the choice between tamoxifen and aromatase inhibitors, which is something we lack in the clinic today.

We use the Danish subcohort of the large randomized Breast International Group (BIG) 1-98 trial of adjuvant tamoxifen versus letrozole (as monotherapy or sequentially) with the aim to investigate AIB1 as a prognostic and predictive biomarker in relation to adjuvant endocrine treatment in estrogen receptor-positive postmenopausal breast cancer.

## Patients and methods

### BIG 1-98

The design of the BIG 1-98 trial, as well as the Danish cohort, has been described in detail before [16–18]. Briefly, this is a randomized, phase 3, double-blinded trial of postmenopausal, estrogen receptor-positive, early breast cancer. Patients were randomized to either monotherapy with tamoxifen or letrozole for 5 years, or to sequential therapy with 2 years of tamoxifen or letrozole followed by an additional 3 years with the other drug (letrozole/tamoxifen). The trial enrolled 1396 Danish patients from 1998 to 2003 included in the intention-to-treat population. Primary tumor samples were available for 1323 of patients and tissue microarrays were constructed for 1281 of these [18–20]. In 1997, the Danish Medicines Agency and the Danish National Committee on Biomedical Research Ethics approved the double-blinded BIG 1-98 trial, and the Ethical Committee of the Capital Region approved the current biomarker study before its activation (KF 02-178/97, KF 12-142/04, RH-2015-166; I-suite 04070). The BIG 1-98 trial is registered on the clinical trial site of the USA National Cancer Institute’s website (http:www.clinicaltrials.gov/ct/show/NCT00004205). The remark criteria were considered for presentation of data below [21].

### Central assessment of the estrogen receptor, progesterone receptor, and HER2

The International Breast Cancer Study Group’s Central Pathology Laboratory carried out a central review on whole tissue sections for estrogen and progesterone receptors by immunohistochemistry, and for *HER2* by immunohistochemistry and fluorescent in situ hybridization as previously described [1, 22]. Tumors expressing estrogen or progesterone receptors in ≥1% of tumor cells were considered hormone receptor positive, and those with a *HER2*-to-Centromere-17 ratio ≥2 considered *HER2*-amplified. The pathology central review was carried out without knowledge of other characteristics, treatment assignment, or outcomes.

### Immunohistochemistry for AIB1

Tissue microarrays were constructed from formalin-fixed and paraffin-embedded tumor blocks by a tissue microarray builder using 2-mm tissue cores [18]. Each tumor was represented by two cores. Immunohistochemistry for AIB1 was performed in an Autostainer-*Plus*, Dako. As a primary antibody for AIB1 detection, a mouse-monoclonal IgG antibody was used at 1:100 dilution (Cat no #611105 BD Bioscience, CA, USA), as previously described [7, 10]. This antibody has been used in several previous clinical trials [3, 7, 8], and its specificity has been confirmed by both Western blot and Northern blot, and in situ hybridization [3, 8]. Immunohistochemical staining (nuclear) was examined by two independent viewers blinded for clinical/tumor characteristics (Sara Alkner and Kristina Lövgren). Each sample was semi-quantitatively scored from 0 to 3 for percentage of stained nuclei and staining intensity. Proportion score 0 represented no stained nuclei, 1:1–10%, 2:11–50%, and 3:51–100%. Staining intensity 0 represented negative staining, 1 weak, 2 moderate, and 3 intense staining. Proportion and intensity scores were added to a total score ranging from 0 to 6. As in several previous publications from our group, a total score of ≥5 was used to define high AIB1 [7, 9, 10, 14]. Cases classified differently (high vs. low AIB1) between viewers (8%) were reexamined to reach consensus. In case of discrepant staining between the two cores from the same patient, the highest score was used.

### Statistical analysis

All clinical data were collected and monitored by the International Breast Cancer Study Group and subsequently transferred to the Danish Breast Cancer Cooperative Group, where the statistical analyses were conducted. Follow-up time was quantified in terms of a Kaplan–Meier estimate of potential follow-up. The primary end-point was disease-free survival, defined as the time from randomization to the first of the following events: recurrence at local, regional, or distant sites; a new invasive cancer in the contralateral breast; any second (non-breast) malignancy; or death without a prior cancer event. Secondary end-point was overall survival, defined as the time from randomization to death, irrespective of cause of death. Time-to-event outcomes were analyzed according to intention to treat. Follow-up was censored at last disease assessment, and in cross-over arms for predictive analysis at 2 years: time of scheduled cross-over.

Baseline characteristics were compared using two-sided Fisher’s exact tests or Wilcoxon rank sum test (age and tumor size). AIB1 expression (low/high) was compared via a stratified log-rank test of disease-free and overall survival, with randomization option (two-arm or four-arm) and treatment arm as a stratification factor, and Kaplan–Meier plots were generated. Cox proportional hazards models were used to estimate hazard ratios and 95% confidence intervals stratified by randomization option; multivariable models were adjusted for age at surgery, tumor size, tumor type and grade, estrogen receptor status, HER2 status, and nodal status. Assumptions of proportional hazard were tested using Schoenfeld residuals and by including a time-dependent component. The interactions of treatment by subpopulation of AIB1 were tested by Cox proportional hazards models including treatment groups, an indicator of the subpopulation, and the interaction term, and likewise for interaction of HER2 and the estrogen receptor by subpopulation of AIB1. Level of statistical significance was set to 5%. Statistical analyses were performed with the SAS v9.4 program package.

## Results

### AIB1 expression and correlation to other tumor markers

**Fig. 1:**
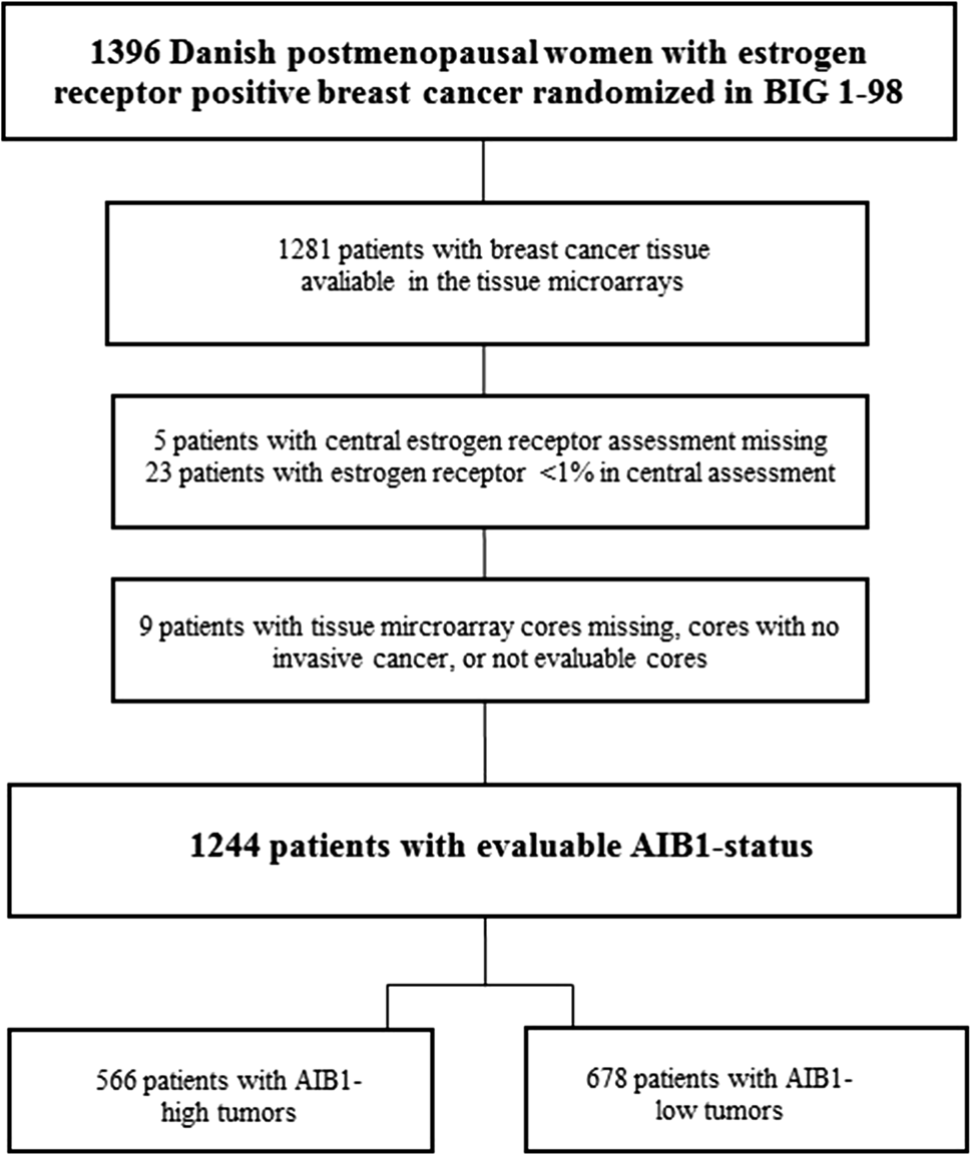
Flow chart of inclusion versus exclusion in the study cohort

Tissue microarray cores from the primary tumor were available from 1281 (92%) of the 1396 Danish participants in the BIG 1-98 trial. Of these, 1244 (97%) were assessable for AIB1. All had estrogen receptor-positive (≥1%) tumors as confirmed by the central assessment. Estimated median potential follow-up was 9.0 years for all patients with full follow-up, and in cross-over arms 7.9 years. There were 440 disease-free survival events and 310 overall survival events. Patient flow and tumor characteristics are described in Fig. 1 and Table 1, respectively. Excluded patients had a higher frequency of small, node-negative tumors and had an earlier year of surgery. A high AIB1 expression was found in 46% of tumors, similar to results from previous studies [9, 10].

**Table 1:** Demographic table, study population

Characteristic	Study population		Excluded		Total		*P* ^a^
	*N*	(%)	*N*	(%)	*N*	(%)	
All	1244	(100.0)	152	(100.0)	1396	(100.0)	
Year of surgery							
1998–99	285	(22.9)	52	(34.2)	337	(24.1)	0.004
2000–01	559	(44.9)	51	(33.6)	610	(43.7)	
2002–03	400	(32.2)	49	(32.2)	449	(32.2)	
Age at randomization (years)							
45–54	148	(11.9)	24	(15.8)	172	(12.3)	0.96
55–64	691	(55.5)	74	(48.7)	765	(54.8)	
65–75	405	(32.6)	54	(35.5)	459	(32.9)	
Tumor size (mm)							
0–20	596	(47.9)	88	(57.9)	684	(49.0)	0.002
21–50	606	(48.8)	62	(40.8)	668	(47.9)	
51+	41	(3.3)	2	(1.3)	43	(3.1)	
Histological type							
Invasive Ductal	1036	(83.3)	132	(86.8)	1168	(83.7)	0.52
Invasive Lobular	173	(13.9)	16	(10.5)	189	(13.5)	
Other	34	(2.7)	4	(2.6)	38	(2.7)	
Tumor grade							
I	269	(25.0)	41	(30.3)	310	(25.6)	0.31
II	639	(59.5)	78	(57.8)	717	(59.3)	
III	166	(15.5)	16	(11.9)	182	(15.1)	
Number of positive lymph nodes							
0	436	(35.1)	69	(45.4)	505	(36.2)	0.04
1–3	531	(42.7)	48	(31.6)	579	(41.5)	
4–9	177	(14.2)	21	(13.8)	198	(14.2)	
≥10	99	(8.0)	14	(9.2)	113	(8.1)	
Estrogen receptor							
<1%	0	(0.0)	23	(44.2)	23	(1.8)	0.62
1–79%	217	(17.4)	6	(11.5)	223	(17.2)	
80–100%	1027	(82.6)	23	(44.2)	1050	(81.0)	
AIB1							
Low	678	(54.5)	14	(53.8)	692	(54.5)	1.00
High	566	(45.5)	12	(46.2)	578	(45.5)	
Score 5	380	(30.6)	8	(30.8)	388	(30.6)	
Score 6	186	(15.0)	4	(15.4)	190	(15.0)	
HER2							
Normal	1118	(91.0)	42	(88.0)	1160	(90.9)	0.44
Amplified	110	(9.0)	6	(13.0)	116	(9.1)	

Unknown values and estrogen receptor values less than 1% were excluded from analysis

^a^Fishers exact test, except for age at randomization and tumor size where Wilcoxon rank sum test was used to compare the distribution of non-aggregated data

*AIB1* amplified in breast cancer 1

High AIB1 expression was associated with a higher tumor grade, *HER2* amplification, high estrogen receptor expression (80–100% vs. 1–79%), a high Ki-67, and ductal histological type (Table 2).

**Table 2:** AIB1 association with demographic and prognostic variables

Characteristic	AIB1						
	Low		High		Total		*P* ^a^
	*N*	(%)	*N*	(%)	*N*	(%)	
All	678	(100.0)	566	(100.0)	1244	(100.0)	
Year of surgery							
1998–99	178	(26.3)	107	(18.9)	285	(22.9)	0.003
2000–01	280	(41.3)	279	(49.3)	559	(44.9)	
2002–03	220	(32.4)	180	(31.8)	400	(32.2)	
Age at randomization (years)							
45–54	72	(10.6)	76	(13.4)	148	(11.9)	0.51
55–64	381	(56.2)	310	(54.8)	691	(55.5)	
65–75	225	(33.2)	180	(31.8)	405	(32.6)	
Tumor size (mm)							
0–20	329	(48.5)	267	(47.3)	596	(47.9)	0.57
21–50	322	(47.5)	284	(50.3)	606	(48.8)	
51+	27	(4.0)	14	(2.5)	41	(3.3)	
Histological type							
Invasive ductal	541	(79.9)	495	(87.5)	1036	(83.3)	0.001
Invasive lobular	111	(16.4)	62	(11.0)	173	(13.9)	
Other	25	(3.7)	9	(1.6)	34	(2.7)	
Tumor grade							
I	184	(32.6)	85	(16.7)	269	(25.0)	<.0001
II	334	(59.2)	305	(59.8)	639	(59.5)	
III	46	(8.2)	120	(23.5)	166	(15.5)	
Number of positive lymph nodes							
0	237	(35.0)	199	(35.2)	436	(35.1)	0.98
1–3	288	(42.5)	243	(42.9)	531	(42.7)	
4–9	99	(14.6)	78	(13.8)	177	(14.2)	
≥10	53	(7.8)	46	(8.1)	99	(8.0)	
Estrogen receptor							0.006
1–79%	137	(20.2)	80	(14.1)	217	(17.4)	
80–100%	541	(79.8)	486	(85.9)	1027	(82.6)	
HER2							
Normal	636	(95.5)	482	(85.8)	1118	(91.0)	<.0001^b^
Amplified	30	(4.5)	80	(14.2)	110	(9.0)	
KI67							
≤14%	356	(54.9)	214	(38.2)	570	(47.1)	<.0001
>14%	293	(45.1)	346	(61.8)	639	(52.9)	
Treatment arm^b^							
Tam	200	(29.5)	164	(29.0)	364	(29.3)	
Let	205	(30.2)	175	(30.9)	380	(30.5)	
T → L	140	(20.6)	116	(20.5)	256	(20.6)	
L → T	133	(19.6)	111	(19.6)	244	(19.6)	

Unknown values and estrogen receptor values less than 1% were excluded from analysis

^a^Fishers exact test, except for age at randomization and tumor size where Wilcoxon rank sum test was used to compare the distribution of non-aggregated data

^b^Exclusion of HER2-amplified cases (as in analyses regarding predictive value of AIB1) only marginally changed the percentage of AIB1-high versus AIB1-low tumors in the respective treatment arm (data not shown)

*AIB1* amplified in breast cancer 1

### AIB1 as a prognostic factor for disease-free and overall survival

**Fig. 2:**
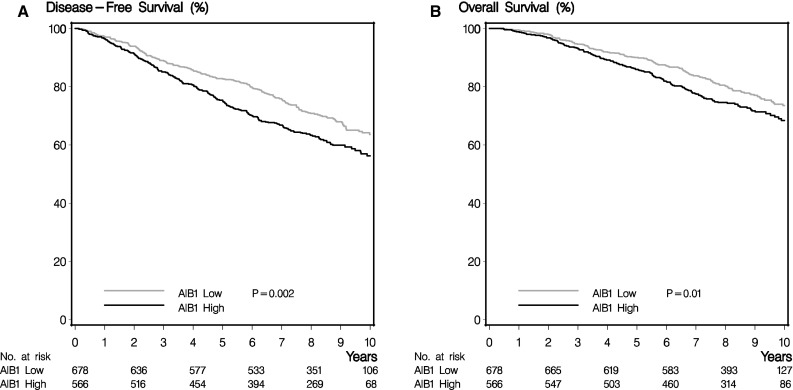
Disease-free survival and overall survival in relation to AIB1. **a** Disease-free survival in relation to AIB1. **b** Overall survival in relation to AIB1

High AIB1 expression was significantly associated with a worse disease-free and overall survival (Table 3; Fig. 2), although for overall survival, significance did not remain in the multivariable analysis. Kaplan–Meier estimates 10 years after randomization showed a disease-free survival of 64% (95% confidence interval 59–68%) for patients with a low AIB1 versus 56% (95% confidence interval 51–61%) for high AIB1. The corresponding numbers for overall survival were 74% (95% confidence interval 69–77%) versus 68% (95% confidence interval 64–73%). An exploratory analysis with a further subdivision of AIB1 into AIB1 score 6 versus score 5 versus score <5 indicated the prognostic effect of AIB1 to be strongest in patients with the highest AIB1 tumor expression (hazard ratio AIB1 score 5: disease-free survival 1.30, overall survival 1.25. Hazard ratio AIB1 score 6: disease-free survival 1.47, overall survival 1.54. AIB1 score <5 as reference. Disease-free survival *P* = 0.005, overall survival *P* = 0.02). All analyses were repeated including only *HER2*-normal and *HER2*-unknown cases with similar results (data not shown). No difference in association between AIB1 and disease-free/overall survival was seen in relation to *HER2* status (disease-free survival *P* = 0.51, overall survival *P* = 0.60).

**Table 3:** Association between AIB1 (high vs. low), disease-free survival (DFS), and overall survival (OS), respectively

Population	Response subgroup	Unadjusted^a^			Adjusted^b^		
		HR	(95% CI)	*P*	HR	(95% CI)	*P*
All patients	DFS	1.35	(1.12–1.63)	0.002	1.29	(1.06–1.58)	0.01
(*N* = 1244)	OS	1.34	(1.07–1.68)	0.01	1.25	(0.99–1.60)	0.07
Only HER2–negative or unknown	DFS	1.27	(1.04–1.55)	0.02	1.28	(1.03–1.57)	0.02
(*N* = 1134)	OS	1.24	(0.97–1.58)	0.08	1.23	(0.95–1.58)	0.11

^a^Cox proportional hazards model stratified for two- or four-arm random assignment option and treatment arm

^b^Cox proportional hazards model stratified for two- or four-arm random assignment option, treatment arm and age at surgery (45–64 vs. 65–75 year). Model further adjusted for estrogen receptor status (1–79 vs. 80–100%), tumor histological grade (I vs. II, and III vs. II), lymph node status, histological type, and tumor diameter

### AIB1 as a predictive marker for treatment with tamoxifen versus letrozole

**Fig. 3:**
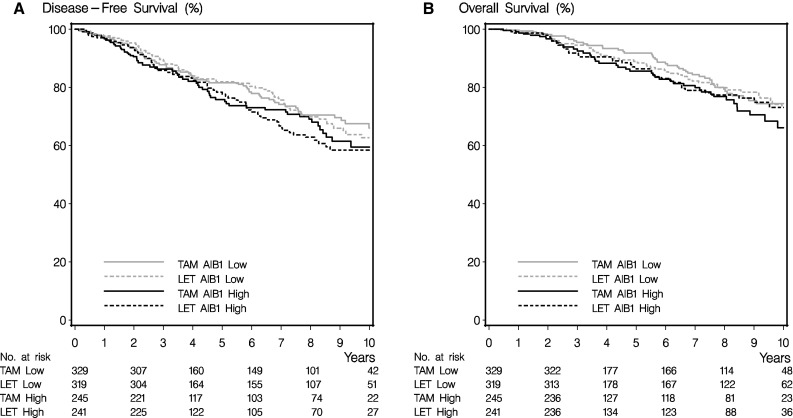
Association between AIB1, treatment (tamoxifen/letrozole), and survival. **a** Disease-free survival. **b** Overall survival. Analyses made in monotherapy arms only (follow-up in cross-over arms truncated 2 years after randomization). *HER2*-amplified tumors excluded

Patients with *HER2*-amplified tumors were excluded, since these patients would not currently be treated with endocrine therapy alone. Hazard ratios for the treatment effect of letrozole versus tamoxifen were as follows—for disease-free survival: AIB1 low 0.85 (95% confidence interval 0.61–1.18) and AIB1 high 1.08 (95% confidence interval 0.76–1.54), and for overall survival: AIB1 low 0.91 (95% confidence interval 0.60–1.37) and AIB1 high 0.91 (95% confidence interval 0.60–1.41). No treatment effect heterogeneity was found between the AIB1-high and AIB1-low populations (Table 4; Fig. 3). Hence, these data indicate a similar benefit of letrozole versus tamoxifen regardless of AIB1 status.

**Table 4:** Treatment effect letrozole versus tamoxifen (*N* = 1134)

Response	Subgroup	Adjusted^a^			
		HR	(95% CI)	*P*	*P*_heterogeneity_^b^
Disease-free survival	All	0.95	(0.75–1.21)	0.68	
	AIB1 Low	0.85	(0.61–1.18)		0.32
	AIB1 High	1.08	(0.76–1.54)		
Overall survival	All	0.91	(0.68–1.23)	0.54	
	AIB1 Low	0.91	(0.60–1.37)		0.99
	AIB1 High	0.91	(0.59–1.41)		

Only HER2-negative or unknown tumors included. Analysis in monotherapy arms only (follow-up in cross-over arms truncated 2 year after randomization)

^a^Cox proportional hazard model stratified for two- or four-arm random assignment option and age at surgery (45–64 vs. 65–75 year). Model further adjusted for estrogen receptor status (1–79 vs. 80–100%), tumor histological grade (I vs. II, and III vs. II), lymph node status, histological type, and tumor diameter

^b^Test for treatment effect heterogeneity

## Discussion

We have investigated the estrogen receptor coactivator AIB1 as a prognostic and predictive factor in relation to endocrine treatment in the Danish cohort of BIG 1-98 (tamoxifen vs. letrozole). We found 46% of tumors to express high levels of AIB1, which is similar to previous publications [9, 10]. In line with earlier studies, AIB1 correlated to a more aggressive tumor phenotype (*HER2* amplification and a high malignancy grade) [5, 7, 8, 23, 24]. In relation to estrogen and progesterone receptor status, results from previous studies have varied. Some show a high AIB1 to be associated with hormone receptor-positive disease [8, 25], while others report an association with estrogen and progesterone receptor negativity [5, 23, 26], or no correlation to receptor status at all [2, 7, 24]. Differences may possibly be explained by different study designs, cut-offs, and study populations. In this cohort, a high AIB1 was associated with a higher estrogen receptor expression.

AIB1 has been reported to be a negative prognostic factor, both in estrogen receptor-positive and estrogen receptor-negative breast cancers [9–11, 14, 24, 26–29]. As a result of studies that show an inferior prognosis for AIB1-high tumors in endocrine-treated estrogen receptor-positive breast cancer cohorts [5, 7, 8], AIB1 has previously been suggested to be of importance for endocrine treatment resistance. However, our earlier investigations of a randomized premenopausal estrogen receptor-positive breast cancer trial (2 years adjuvant tamoxifen vs. control) clearly indicate that although AIB1-high tumors had an inferior prognosis early on, these patients responded very well to tamoxifen [10]. AIB1-low tumors had a better prognosis early on, but this was not further improved by tamoxifen. Importantly, these results have been confirmed in a randomized tamoxifen trial including postmenopausal estrogen receptor-positive disease [15]. Hence, we hypothesize that the association between a high AIB1 expression and poor prognosis is related to its prognostic significance, which cannot be entirely eradicated by adjuvant endocrine treatment. The strong prognostic effect of AIB1 makes it a very interesting target for new anti-cancer therapies. Although steroid receptor coactivators are large unstructured proteins making production of drugs against them challenging, there are ongoing efforts to pharmacologically target them [30, 31].

Today there are no markers that predict which population of postmenopausal estrogen receptor-positive breast cancer patients that are likely to have superior benefit from tamoxifen versus aromatase inhibitors. Due to AIB1’s predictive effect in relation to tamoxifen, we postulated that AIB1 status might be a useful marker. Previous trials regarding AIB1’s relation to aromatase inhibitors are very sparse, include small cohorts, and show conflicting results [12, 13]. The data presented here are, to our knowledge, the first time AIB1 is investigated in relation to aromatase inhibitors in a large randomized clinical trial. However, we found no evidence of differences in treatment effect between tamoxifen and letrozole in relation to AIB1 status. Hence, our study indicates that tumor expression of AIB1 cannot be applied as a predictive marker for selection of tamoxifen versus letrozole as adjuvant therapy in postmenopausal endocrine-responsive breast cancer.

A relationship between AIB1 and HER2 has previously been suggested, with a worse prognosis with co-expression of AIB1 and HER2 [5, 8, 32]. We found a correlation between a high AIB1 and *HER2* amplification. However, in line with our previous studies, no significant interaction between AIB1 and HER2 in relation to prognosis was detected [9, 14]. As in all earlier studies though, AIB1-high, *HER2*-amplified tumors represented only a small subgroup of the cohort.

Although we had the advantage of using a large international controlled randomized trial, this study still has some potential limitations. Most importantly, the BIG 1-98 trial did not include a control group of patients not receiving endocrine therapy. Access to such a group would probably have clarified the prognostic and predictive value of AIB1 even more, especially in relation to letrozole. In addition, after an interim analysis showed a superior effect for letrozole, patients randomized to tamoxifen were allowed a treatment switch, reducing the possibility to detect differences in treatment effect [17]. Furthermore, although a large patient cohort is included, as in all studies, numbers are strongly reduced in subgroup analyses, such as investigations of AIB1-high/*HER2*-amplified tumors. Finally, although we used a cut-off to define a high AIB1, which has been used in several previous studies, AIB1 is still an explorative biomarker and an optimal cut-off is yet to be definitely determined.

In conclusion, in a subset of the BIG 1-98 study population, we confirm tumor expression of AIB1 to be a strong negative prognostic factor. As the association with a high AIB1 and poor prognosis has now been repeatedly shown in different patient cohorts, AIB1 is an interesting target for anti-cancer therapies. However, no difference in treatment effect between tamoxifen and letrozole in relation to AIB1 was found. Hence, AIB1 cannot be of assistance for the choice of type of endocrine treatment in postmenopausal endocrine-responsive disease.
